# Sweet Basil (*Ocimum basilicum* L.)―A Review of Its Botany, Phytochemistry, Pharmacological Activities, and Biotechnological Development

**DOI:** 10.3390/plants12244148

**Published:** 2023-12-13

**Authors:** Nabilah Sekar Azizah, Budi Irawan, Joko Kusmoro, Wahyu Safriansyah, Kindi Farabi, Dina Oktavia, Febri Doni, Mia Miranti

**Affiliations:** 1 Department of Biology, Faculty of Mathematics and Natural Sciences, Universitas Padjadjaran, Jatinangor 45363, Indonesia; nabilah17004@mail.unpad.ac.id (N.S.A.); budi.irawan@unpad.ac.id (B.I.); joko.kusmoro@unpad.ac.id (J.K.); febri@unpad.ac.id (F.D.); 2 Department of Chemistry, Faculty of Mathematics and Natural Sciences, Universitas Padjadjaran, Jatinangor 45363, Indonesia; wahyu17002@mail.unpad.ac.id (W.S.); kindi.farabi@unpad.ac.id (K.F.); 3 Department of Transdisciplinary, Graduate School, Universitas Padjadjaran, Bandung 40132, Indonesia; dina.oktavia@unpad.ac.id

**Keywords:** *Ocimum basilicum* L., antiviral, antifungal, anticancer, nanotechnology, biotechnology

## Abstract

An urgent demand for natural compound alternatives to conventional medications has arisen due to global health challenges, such as drug resistance and the adverse effects associated with synthetic drugs. Plant extracts are considered an alternative due to their favorable safety profiles and potential for reducing side effects. Sweet basil (*Ocimum basilicum* L.) is a valuable plant resource and a potential candidate for the development of pharmaceutical medications. A single pure compound or a combination of compounds exhibits exceptional medicinal properties, including antiviral activity against both DNA and RNA viruses, antibacterial effects against both Gram-positive and Gram-negative bacteria, antifungal properties, antioxidant activity, antidiabetic potential, neuroprotective qualities, and anticancer properties. The plant contains various phytochemical constituents, which mostly consist of linalool, eucalyptol, estragole, and eugenol. For centuries, community and traditional healers across the globe have employed *O. basilicum* L. to treat a wide range of ailments, including flu, fever, colds, as well as issues pertaining to digestion, reproduction, and respiration. In addition, the current research presented underscores the significant potential of *O. basilicum*-related nanotechnology applications in addressing diverse challenges and advancing numerous fields. This promising avenue of exploration holds great potential for future scientific and technological advancements, promising improved utilization of medicinal products derived from *O. basilicum* L.

## 1. Introduction

In recent years, natural plant-based products have emerged as a valuable global resource for the development and innovation of novel drugs [1,2]. Hence, exploring bioactive compounds from various sources, including plants, might be an excellent method for discovering new potential drugs [3]. This is because the current availability of raw materials for drug discovery and development, pharmacophores, and a framework for effective medications for a wide range of clinical indications is notably limited [4]. Hence, ethnopharmacological studies are of great significance, as they harness traditional knowledge to effectively screen and improve the chances of discovering novel drugs [5].

Basil (*Ocimum basilicum* L.) is one of the species in the Lamiaceae family, which is well known for having a wide variety of medicinal properties [6]. The plant is traditionally recognized for its utilization for both culinary and perfumery purposes [7]. For example, in the province of East Nusa Tenggara, Indonesia, the Tetun people frequently consume fresh, raw *O. basilicum* L. leaves in order to treat malaria [8]. In addition, it is also used for treating rheumatism, high cholesterol, hypertension, headaches, and stroke in the Indonesian province of North Sumatra by the Batak Karo people [9]. *O. basilicum* L. leaves also find application as an anti-helminthic remedy among the Muna Tribe in the province of Southeast Sulawesi, Indonesia [10].

Some of the uses stated are associated with the main constituents found in *O. basilicum* L. plant parts, which include linalool, eugenol, geranial, methyl eugenol, 1,8-cineole, and other compounds [11]. These compounds were found to play important roles as antimicrobials, antioxidants, anticancer agents, and antidiabetics [12]. Certain chemical compounds, specifically linalool and eugenol, are in great demand at present. This urgency arises from the resistance exhibited by *Staphylococcus aureus*, which is known for its ability to create biofilms [13].

This article aims to present a comprehensive overview of the current and ongoing progress in the use of *O. basilicum* L. for medical purposes in human and animal healthcare, with the aim of serving as a guide, which traces the historical uses of *O. basilicum* L. from ethnopharmacology to biotechnological development. Additionally, this article aims to promote further clinical research efforts and the development of pharmaceutical formulations using *O. basilicum* L. as a valuable resource.

The investigation commences by examining the morphological and chemical compositions of *O. basilicum* L. Subsequently, we proceed to gather empirical evidence derived from ethnomedicinal data originating from diverse regions and continents around the world. Moreover, there are substantial data supporting the therapeutic benefits of this plant species from the perspectives of microbiology and biomedicine. Finally, we will explore the future prospects of nanotechnology in this field and investigate the strategies to enhance its metabolite production.

## 2. Methodology

A literature survey using various keywords, such as “*Ocimum basilicum* L.”, “antiviral”, “phytochemical constituents”, “ethnomedicinal use”, was conducted in scientific databases, including Scopus, ResearchGate, and Google Scholar. Out of all the collected publications, 156 underwent thorough evaluation and included research articles, review articles, and book chapters. From this array of scientific resources, our focus was directed toward the antimicrobial activity, phytochemical constituents, and biotechnological advancements of *O. basilicum* L.

## 3. *Ocimum basilicum* L. Ecology and Morphology

Basil is renowned for its ability to thrive in diverse temperature ranges and geographical regions, making it a globally cultivated herb [14]. The genus *Ocimum*, which belongs to the Lamiaceae (Labiatae) family, has distribution throughout tropical and subtropical America, Africa, and Asia continents [15]. *Ocimum* has over 150 species and is extensively cultivated in countries such as Indonesia, India, Morocco, France, Hungary, Greece, and Egypt [16]. Although it is grown as a common garden herb, basil is most likely native to Asia and Africa. It is believed that Alexander the Great (356–323 BCE) brought it from India to ancient Greece, to England in the middle of the 1500s, and to the United States in the early 1600s. Many countries, including Egypt, India, Indonesia, Mexico, and the United States, produce this plant commercially for the market [17].

Sweet basil is an annual herb with dense foliage and a variety of aromatic components [18]. This plant thrives in an agroclimatic environment, with temperatures ranging from 7 to 27 °C, annual precipitation from 0.6 to 4.3 m, and soil pH from 4.3 to 8.2. This plant requires low maintenance, and it is easy to grow in indoor and outdoor settings [17]. Although it can be damaged by frost and temperatures below freezing, this species flourishes under conditions of long daylight with full sun and well-drained soil [19].

**Figure 1:**
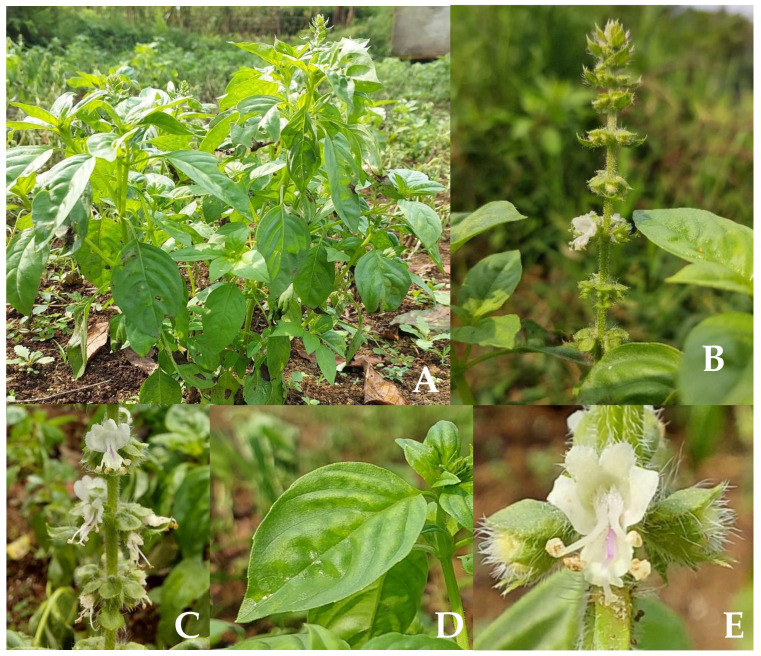
(**A**) *O. basilicum* L. plant habit in a plantation area of Parongpong subdistrict, Bandung, Indonesia. (**B**) Full *O. basilicum* L. inflorescence. (**C**) Separate photograph detailed section of inflorescence. (**D**) Leaf. (**E**) Flower (photographs courtesy of Nabilah Sekar Azizah).

The plant can grow up to 0.6 m in height, with lateral branches creating an angle of more than 30° with the main branch. The stem is round–quadrangular, glabrous (smooth, hairless), or puberulent (fine short hairs), concentrated on the two opposing faces of the stem (Figure 1A). Inflorescence is dense (Figure 1B), arranged around a point on an axis up to 12 mm apart; the axis is pubescent and with a total of six flowers surrounding the apex (Figure 1C). The leaves are green, the apex mostly acute or acuminate; the shape is ovate or elliptic ovate; the size is about 15–50 × 5–25 mm; the leaf margin is entirely or sparsely serrate and with a glandular–punctate shape. The petiole is about 20 mm long and pubescent (covered with soft short hair) (Figure 1D). The corolla with a white or pinkish color tube—about 7–8 mm long—is funnel-shaped (Figure 1E). The calyx pilose (covered with soft long hair or pubescent) has a dense ring of hairs at the throat, and a fruiting calyx is about 6 mm long. The stamen has tufted hairs near the base. The nutlets are dark brown in color, with an elliptic shape, and they produce mucilage upon interaction with water [20,21].

## 4. Phytochemical Constituents

The type of chemotype can affect the main chemical constituent of *O. basilicum* L. [22]. Varga et al. discovered five chemotypes, among which (A) linalool (**15**); (B) linalool (**15**)/*trans-α*-bergamotene (**29**); (C) linalool (**15**)/methyl chavicol (**21**); (D) linalool (**15**)/*trans*-methyl cinnamate (**25**); and (E) methyl chavicol (**21**). Based on the distribution in the regions, chemotypes A and C are European chemotypes; D is a tropical chemotype; and E is a Reunion chemotype [22]. The chemical constituents of *O. basilicum* L. were dominated by compounds from the phenylpropanoid and monoterpenoid classes [23].

One of the most crucial aspects determining the quality of essential oil depends on the method by which it was adopted. In some studies, conventional extraction methods, such as Soxhlet, hydrodistillation, steam distillation, solvent extraction, and a combination of steam and solvent extraction, are still being used [24]. However, there are promising green extraction methods, including microwave-assisted extraction (MAE), ultrasound-assisted extraction (UAE), high-pressure-assisted extraction (HAE), supercritical and subcritical fluid extraction, electrically assisted extractions, and enzyme-assisted extraction [25]. Green extraction techniques address the challenges by offering several advantages, including cutting down on the use of organic solvents, ease of use due to the simplicity, time efficiency, and cost effectiveness in the extraction process [26].

The *O. basilicum* L. extract and essential oil contain classes of chemical compounds, mainly terpenoids, such as oxygenated sesquiterpenes, oxygenated monoterpenes, sesquiterpene hydrocarbons, monoterpene hydrocarbons, and non-terpene derivatives. Furthermore, this plant contains phenylpropanoid compounds, including eugenol, methyl eugenol, chavicol, estragole, and methyl cinnamate [27,28]. Monoterpene, geraniol, myrtenol, pinene, camphor, and borneol hold potential for medical applications [29].

The analysis of *O. basilicum* L. methanolic leaves’ extract, obtained by sonication, showed the presence of various polyphenol compounds, such as caffeic acid, caftaric acid, 3,4-dihydroxyphenylacetic acid, ferulic acid, rosmarinic acid, and rutoside (rutin) [30]. Phenolic compounds have been widely recognized for their beneficial properties and applications in the medical field [31]. Furthermore, *O. basilicum* L. extract was also found to contain phytosterols, such as β-sitosterol, stigmasterol [32], and campesterol [33].

**Figure 2:**
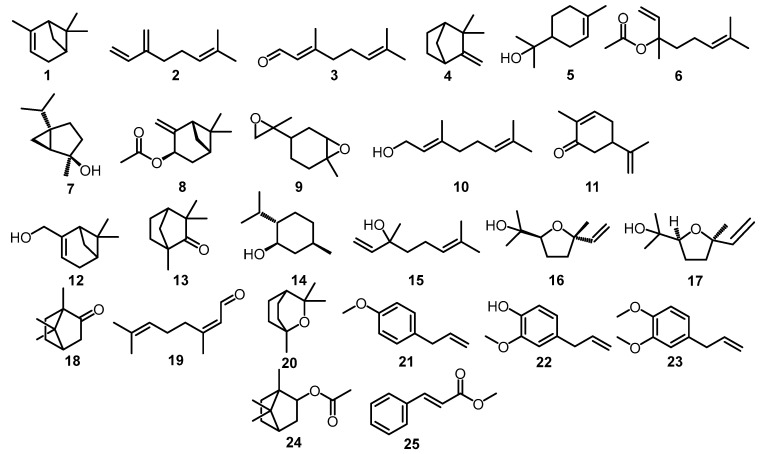
Structure of monoterpenoids.

**Figure 3:**
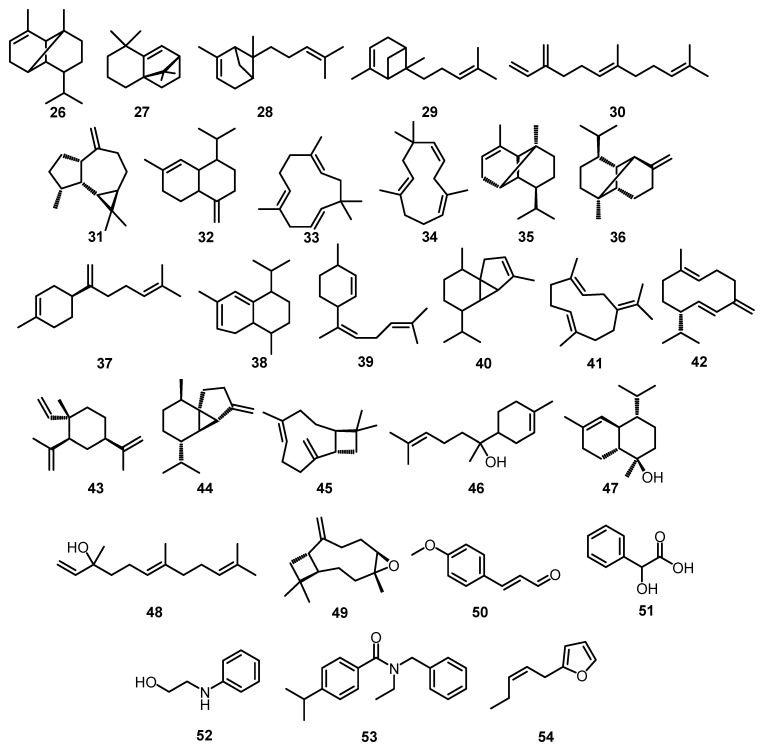
Structure of sesquiterpenoids.

Studies on the chemical constituents of *O. basilicum* L. are shown in Table 1, representing its essential oil and extract with various extraction and identification methods. Meanwhile, Figure 2 and Figure 3 show the chemical structures of the chemical compounds presented in Table 1.

## 5. Ethnomedicinal Evidence for *O. basilicum* L.

More than 10% of plant species and over 50,000 species have been utilized for the development of medications and healthcare products [2]. *O. basilicum* L., also known as the king of herbs in the Greek word, has been recognized since ancient times for its therapeutic properties and was used in the Unani and Ayurvedic medical systems [41]. The proper way to use medicinal plants is typically passed down from one generation to the next and often pertains to traditional remedies for age-old ailments [42]. These traditional beliefs about medicinal plants will be enhanced by the integration of technology for the production of sustainable pharmaceuticals.

Ethnobotany is a subdiscipline of ethnobiology, which studies the traditional botanical knowledge in different cultures, the techniques for utilizing plants, the management of plant resources, and the role, which plants play in ritual, cultural, or religious beliefs [43]. As a result, it serves as a foundation for selecting plants, which can be developed for medicinal purposes [44]. Ethnobotany contributes to exploration of the ways to fill the gap between scientific research and cultural or indigenous understanding [45]. The majority of ethnobotanical and ethnopharmacological studies have been conducted to acquire knowledge about the use of medicinal plants to treat various illnesses [46].

India is known as a country for the knowledge and utilization of herbal medicine [47]. Indigenous and local people in the Bageshwar District of Uttarakhand, India, use *O. basilicum* L. leaf and seed for treating fever, cough, and cold [48]. The community around Lawachara National Park in Bangladesh utilizes *O. basilicum* L. leaf as a treatment for reducing high blood pressure, fever, and cough [49]. Another area in Lalmohan, Bhola District, Bangladesh, utilized the whole plant of *O. basilicum* L. to treat fever and as a carminative [50].

Remote areas in India, such as the Uttara Kannada District, used herbal treatments with *O. basilicum* L. to treat reproductive diseases, such as dysmenorrhea, by crushing the bark in milk and drinking it once a day for seven days [51]. Additionally, local healers from the Khatling Valley and Pauri District in Uttarakhand, India, drink the decoction of leaves and seeds of *O. basilicum* L. for fever, cough, cold, and urinary problems [52,53]. Traditional healers in the North West Ganjam District, Odisha, India, employ powder and decoction from the leaves of *O. basilicum* L. for treating dysuria, cough, and cold [54]. Meanwhile, the traditional healers from the Rabha Tribe in the Kamrup District, India, use the leaves and inflorescence as a remedy for cough and chronic fungal infections [55].

Pakyoung in East Sikkim, India, reported that the leaves and seeds of *O. basilicum* L. were utilized for colds, coughs, fevers, and constipation [56]. Based on an interview with 33 traditional healers from Nelliyampathy, Kerala, India, the leaves and seeds of *O. basilicum* L. were made into paste, inhalation, juice, and infusion, which could treat tumors, headaches, insomnia, heart trembling, coughs, chest pain, dysentery, diarrhea, and gonorrhea [57].

In Dharan, Nepal, *O. basilicum* L. leaf juices are used for digestive disorders, such as diarrhea, dysentery, constipation, gastritis, and vomiting [58]. In the Sulaymaniyah province in Iraq, it had been reported that the local community applied *O. basilicum* L. as an ethnobotanical treatment for headaches, colds, and halitosis (bad breath) [59]. Meanwhile, in the Peshawar Valley, Pakistan, *O. basilicum* L. leaves were made into an extract and given orally to help improve digestion and for other purposes, such as ornamental decoration [60].

*O. basilicum* L. has a history of being used as a medical treatment in some southeast Asian nations for a variety of diseases. Traditional healers from Phatthalung, southern Thailand, use *O. basilicum* L. for treating flatulence and peptic ulcers [61]. Local tribes from Tina and Libas Gua Village, Mindanao, Philippines, utilized the leaves by rubbing them around the affected area of the body to treat cold sores [62]. Furthermore, in Indonesia, this plant is widely used in the big islands, such as Sulawesi, Sumatra, and Kalimantan. The tribes in Kolaka and East Kolaka, Southeast Sulawesi, make use of the leaves of *O. basilicum* L. for treating tuberculosis [63]. Meanwhile, the traditional healers in many areas of Sumatra utilized the seeds of *O. basilicum* L. as a therapy for treating back pain sickness [64]. The sub-ethnic Dayak tribe—like the Dayak Linoh tribe, who live in the Sintang District, West Kalimantan—utilized the leaves, flowers, and fruit to reduce body odor and fever [65]. Another sub-ethnic group, Dayak Tamambaloh, in the Kapuas Hulu District, West Kalimantan, used the leaves to treat ringworm, blisters, and reduce body odor [66].

In addition, in the South American continent, e.g., in Maragogipe, State of Bahia, Brazil, the leaves were made into tea for the treatment of delayed menstruation, fever, flu in children, indigestion, and nasal congestion [67]. In an ethnomedicine inventory record in the African continent, e.g., the Abia State in Nigeria, the seeds of *O. basilicum* L. were used for treating diarrhea [68]. In Western Oromia State, Ethiopia, this species is used to cure allergic reactions by crushing it and mixing it with food [69]. In central Kenya, the plant is used for curing common cold [70]. In the Rainforest Research Station, Ondo State, Nigeria, the whole plant of *O. basilicum* L. is used for treating inflammation [71].

The utilization of *O. basilicum* L. as a herbal remedy has been observed on a global scale and spread across regions such as Asia, Africa, and South America. Traditional healers from various regions used the whole plant, as well as some parts of the plant, such as leaves, seeds, inflorescences, fruits, and stems. The plants were made into infusion, inhalation, paste formulations, powder, tea, and they were incorporated into food. According to the beliefs of traditional healers, *O. basilicum* L. possesses therapeutic properties in the management of typical illnesses affecting the digestive, respiratory, urinary, and reproductive systems. Additionally, certain individuals within the society employed this particular plant for the purpose of enhancing decorative esthetics.

## 6. Antimicrobial Activities and Biomedical Uses

Medicinal plants contain rich, yet underexploited, bioactive compounds, with a limited amount of their potential qualities having been thoroughly examined [72]. The exploration of a medicinal plant with high chemical constituents holds promise in the pursuit of developing therapeutic treatments in the future [73]. Each part of the plant offers potentially valuable biomedical knowledge, which remains uncovered [74]. As one of the medicinal plants, *O. basilicum* L. possesses considerable undiscovered potential in the field of antimicrobial and biomedical research. The goal of biomedical therapy through the utilization of medicinal plants requires continuous exploration and development.

### 6.1. Antiviral Activity

Based on the ethnomedicinal records and data, traditional healers all over the world consider *O. basilicum* L. to be an important herb. A number of studies have shown that *O. basilicum* L. has the potential for antiviral activity. For instance, a recent study of *O. basilicum* L. against SARS-CoV-2 with an in silico assay showed that polyphenol constituents apigenin-7-glucuronide and dihydrokaempferol-3-glucoside have binding affinity for −8.77 Kcal/mol and −8.96 Kcal/mol, respectively, which possess great potential for antiviral activity. These compounds have binding affinity with the main protease (M^pro^) enzymes on SARS-CoV-2 [75]. The M^pro^ enzyme, typically referred to as 3-chymotrypsin-like protease (3CL^pro^), serves a pivotal role in viral replication and is being targeted as a way of preventing COVID-19 infection [76].

Aside from the polyphenol compound, for the first time, monoterpenes showed the potential for antiviral activity toward SARS-CoV-2. An in vitro study demonstrated that five different monoterpene compounds, such as carvone, carvacrol, menthofuran, 1,8 cineole, and pulegone, potentially inhibited SARS-CoV-2 in infected Vero 76 cells. Among these five compounds, carvacrol and carvone showed significant antiviral activity with half-maximal inhibitory concentration (IC_50_) < 100 µM. In addition, an essential oil, which contained the highest carvone concentration (>200 mg/mL), had the greatest antiviral activity with IC_50_ 127 ± 4.63 ppm. The antiviral properties of monoterpene compounds had been observed to bind and interrupt the important viral proteins, among them, the main protease and spike protein [77]. On top of its potent antiviral effects, carvacrol had a favorable safety profile, suggesting its potential as a viable candidate for the development of preventive therapies [78].

Another recent study tested the lipophilic fraction of the stem of this plant in vitro and in silico against dengue virus (DENV). This in vitro study revealed that the fraction significantly reduced DENV titer in pre-treatment and post-treatment conditions at a concentration of 3.125 µg/mL. Meanwhile, an in silico study of the lipophilic fraction showed that the two compounds had great binding affinity. Stigmasterol had a binding affinity for −8.3 Kcal/mol with NS1 protein, and campesterol exhibited the biggest binding affinity for −8.2 Kcal/mol with E glycoprotein [33]. A computational study demonstrated that this plant has the potential for antiviral drug development through its mechanism of inhibiting the active site of human immunodeficiency virus (HIV) gp120 and gp41. α-guaiene is the compound with the highest negative value for binding affinity, which was −9.62 Kcal/mol at the active site gp120, and sitosterol displayed a binding affinity for −10.99 Kcal/mol at the active site gp41 [79]. Furthermore, an in vitro study of the crude ethanolic extract of *O. basilicum* L. leaves demonstrated antiviral activity against Zika virus (ZIKV) with 97% virus infectivity at the highest concentration (1:16 dilution) [80].

There are a few studies, which show that *O. basilicum* L. can treat some viruses infecting livestock, such as cattle and poultry. An in vitro study of alcoholic extract from *O. basilicum* L. leaves showed potential in managing the Newcastle disease virus (NDV), which infects poultry, with a reduction titer up to 10^−7^ at the concentration of 500 µg/mL [81]. Kubiça et al. reported an in vitro study of 1,8-cineole and camphor, which both reduced the plaque in bovine viral diarrhea virus (BVDV) by approximately 75% and 84%, respectively, at the maximum non-toxic dose [82]. Another ethanolic extract of *O. basilicum* L. was made into an ointment for daily application in bovine cutaneous papillomatosis, which is caused by bovine papillomavirus (BV). The ointment was made into a 2% formulation with 20 mg/g weight per weight (*w*/*w*) of ethanolic extract and white petroleum jelly for the base. Clinically, the papilloma began to regress and eventually disappeared between days 7 and 21, and the skin texture progressively returned to normal. The antiviral activity of this topical formulation may be due to the phenolic, flavonoid, tannin, and alkaloid compounds found in it [83].

Another crude aqueous and ethanolic extracts of the whole plant of *O. basilicum* L., as well as selected purified constituents, exhibited antiviral activity against DNA viruses, such as the herpes virus (HSV), adenoviruses (ADV), hepatitis-B virus (HBV), and RNA viruses, such as coxsackievirus B1 (CVB1) and enterovirus 71 (EV71) [84]. This in vitro research found that purified constituents, such as apigenin, ursolic acid, and linalool, had antiviral activity against HSV-1 similar to that of acyclovir and also against HBV and enterovirus. The strongest purified constituents were ursolic acid against HSV-1 (EC_50_ 6.6 mg/L), ADV-8 (EC_50_ 4.2 mg/L), CVB1 (EC_50_ 0.4 mg/L), and EV71 (EC_50_ 0.5 mg/L). Furthermore, apigenin possessed the highest antiviral activity against HSV-2 (EC_50_ 9.7 mg/L), ADV-3 (EC_50_ 11.1 mg/L), and hepatitis-B surface antigen (EC_50_ 7.1 mg/L). Meanwhile, linalool has recorded moderate anti-adenoviral activity against ADV-3, ADV-8, and it has exhibited the strongest effects against ADV-11 (EC_50_ 16.9 mg/L) [84]. In addition, methanolic extracts of *O. basilicum* L. significantly inhibited herpes simplex virus 1 strain F (HSV-1F) after viral adsorption [85]. Another purified compound, such as eugenol, from the methanolic extract of *O. basilicum* L. showed inhibition of pre-HIV-1 infection in the host cell at the effective concentration of 350 µg/mL [86].

The evidence presented above indicates that the phytochemical constituents of *O. basilicum* L. hold the potential to be developed into new drugs, offering an opportunity to address issues such as drug resistance and side effects [87]. Understanding the phytochemical constituent mechanisms of action in pharmacology is crucial before developing drugs from *O. basilicum* L. [88]. The antiviral properties of phytochemicals have been subject of substantial research in recent years, even after the world experienced a worldwide pandemic [89]. Therefore, it is crucial to maintain and expand this research into their antiviral properties in light of ongoing global health challenges.

### 6.2. Antibacterial Activity

The increasing incidence of antibiotic resistance in recent years has prompted an immediate demand for new strategies and innovative antibiotic formulations [90]. The aforementioned issue arises from the irresponsible use of antibiotics in the context of human healthcare and the practice of animal husbandry [91]. *O. basilicum* L. is one of many medicinal plants, which have demonstrated potential as antibacterial agents. Essential oils, methanolic extracts, and fractions from the plant have been explored over these past few years for their antibacterial properties against Gram-positive and Gram-negative bacteria. The evidence of antibacterial activity against Gram-positive and Gram-negative bacteria is shown at the minimum inhibitory concentration (MIC) in Table 2, and the diameter of the inhibitory zone is shown in Table 3.

An in vitro study of the antibacterial characteristics of *O. basilicum* L. essential oil demonstrated inhibition and eradication activities against *Vibrio* strains’ mature biofilm. A concentration of 50 mg/mL of *O. basilicum* L. essential oil was shown to greatly inhibit the biofilm, with a percentage of 55% for *V. parahaemolyticus* and up to 87.45% for both *V. vulnificus* and *V. cholerae*. The bactericidal effects of this essential oil may have a correlation with the high amount of linalool found in its composition [99]. The potential antibacterial activity of monoterpene compounds involves the degradation of cell walls and cell membranes, as well as the interruption of membrane protein and ion transport processes [100].

Another study demonstrated that methanolic leaf extracts of *O. basilicum* L. were shown to have a great potential in antibacterial activity against *B. cereus*, *P. aeruginosa*, *L. monocytogenes*, *E. coli*, *M. flavus*, and *S. aureus,* with MIC < 0.5 mg/mL and MBC < 0.9 mg/mL. This methanolic extract contains some polyphenol compounds, such as 3,4-dihydroxyphenylacetic acid and rutoside (rutin), which mainly contribute to the antibacterial effects [30]. The antibacterial effects of polyphenol compounds arise from their ability to interact with bacterial cell walls and membranes, disrupt protein regulation, inhibit microbial enzymes, and exhibit iron-chelating capabilities [101].

The essential oil of *O. basilicum* L. combined with the antibiotic imipenem had a synergistic interaction, resulting in antibacterial activity against *S. aureus* and *P. aeruginosa*. Meanwhile, the essential oil combined with the antibiotic ciprofloxacin had an antagonistic and indifferent interaction [95]. This means that *O. basilicum* L. phytochemical-derived substances have the potential to enhance the existing antibiotics. Anwar et al. demonstrated that *O. basilicum* L. in multiple regions of Saudi Arabia exhibited variations in antibacterial activity because of the different chemical compositions, which are influenced by the diverse agro-climatic regions [102].

In addition to diverse regions, various factors influence antimicrobial activity, such as the chemical compounds, bacterial strain, temperature, and bacterial cell number [103]. The evidence of antibacterial properties indicates that *O. basilicum* L. has a broad spectrum of antibacterial activity against both Gram-positive and Gram-negative bacteria.

### 6.3. Antifungal Activity

In the intensive care unit (ICU), invasive fungal infections are commonly identified in patients with a weakened immune system [104]. Fungal infections are a major cause of infectious-disease-related mortality around the world [105]. Additionally, because fungi are eucaryote organisms, they only have a few molecular targets, which can be exploited by medications to activate their effects [106]. Recently, phytochemical constituents have received a lot of interest in the research and development of antifungal medications.

The essential oil of *O. basilicum* L. exhibits antifungal activity against pathogenic fungal *Aspergillus flavus* at a concentration of 1000 ppm, which could suppress the fungal growth and aflatoxin B1 biosynthesis. The chemical compounds, which possess good antifungal properties, are linalool and 1,8-cineol from monoterpene and eugenol from polyphenol [40]. The commercial basil extracts in Slovenia were tested against several *Fusarium* species for antifungal properties. The extracts were found to inhibit colony growth in *F. proliferatum* at concentrations of, respectively, 0.35% and 0.70% by up to 33.37% and 44.30%, and they inhibited colony growth in *F. subglutinans* by up to 24.74% and 29.27%. The commercial extracts are known to contain estragole (86.72%), *trans-α*-bergamotene (2.91%), and eucalyptol or 1,8-cineole (2.67%) [107].

The essential oil of *O. basilicum* L. has antifungal properties against *Candida albicans,* with an inhibition zone of 27 mm. These activities are supported by linalool, methyl chavicol, β-elemene, and α-bulnesene [108]. In Serbia, twelve cultivars of *O. basilicum* L. were tested against seven species of fungi, including *A. ochraceus*, *A. versicolor*, *A. fumigatus*, *A. niger*, *Penicillium funiculosum*, *P. ochrochloron*, and *Trichoderma viridae*. The twelve cultivars exhibited great antifungal properties in inhibiting fungal growth, with the minimum inhibition concentration being 10–100-fold higher than the commercial antifungal drug ketoconazole and minimum fungicidal concentrations ranging from 0.14 µg/mL to 27.00 µg/mL. These antifungal properties of the twelve cultivars of *O. basilicum* L. may be due to high linalool composition in the essential oils [109].

In addition to essential oil, the methanolic fraction from the aerial parts of *O*. *basilicum* L. showed antifungal activity. The fraction was tested against eight species of fungi, including *A. flavus*, *A. niger*, *Penicillium*, *Rhizopus solani*, *Alterneria alternata*, *Candida albicans*, *Curvilaria lunata*, and *A. fumigates*. These methanolic fractions exhibited strong inhibition of fungal growth, from the lowest at 10% up to 100%, at concentrations of 1 mg/mL, 3 mg/mL, and 6 mg/mL. *C. albicans* was one of the resistant species; at 3 mg/mL, it only had 17% inhibition compared to other species, which had 43–100% inhibition [110]. In contrast, the ethanolic extracts from the aerial parts of *O. basilicum* L. demonstrated antifungal activities against *C. albicans*. The extracts showed the presence of an inhibitory zone measuring 18 mm for the fungi [111].

A study showed that high concentrations of terpene compounds, such as citral, eugenol, nerolidol, and α-pinene, demonstrated an antifungal mechanism by breaking down the cell membrane [112]. Meanwhile, an in silico study of polyphenols from the plant, including rutin, kaempferol, and quercetin, demonstrated an underlying antifungal mechanism. The polyphenol compound was found to have the ability to inhibit fungal enzymes. Rutin—also called rutoside—had the greatest antifungal activity through binding with 14-alpha demethylase (CYP51) and nucleoside diphosphokinase (NDK), with binding affinity for −9.4 and −8.9, respectively [113].

The synergistic action of various constituents comprising the essential oil suppresses the chance of resistance. This is attributed to the difficulty the pathogens face in adapting resistance characteristics against multiple compounds present in essential oils [114]. This indicates that *O. basilicum* L. has potential for development into a new class of natural antifungal drugs.

### 6.4. Biomedical Activity

Numerous records and findings have demonstrated the advantages of *O. basilicum* L. plant in the medical field, such as its antioxidant, anticancer, analgesic, antidiabetic, anti-inflammatory, and antidepressant effects [12]. The *O. basilicum* L. essential oil from the seeds had good antioxidant activity when using the DPPH assay compared to the positive control, which was the Trolox compound. Great antioxidant activity was shown with an inhibition concentration (IC_50_) of 23.44 ± 0.9 µg/mL [115]. Antioxidant activity eventually resulted from the synergy of each compound and had correlation with the total phenolic compound, which composed the essential oil [116]. The phenolic compound has the ability to donate a hydrogen atom to the free radicals, the ability to chelate metal cations, and the ability to scavenge free radicals [39,117]. Stanojevic et al. also reported that with the DPPH assay, basil essential oil had good antioxidant properties with effective concentration (EC_50_) at 2.38 ± 0.10 mg/mL, and it could be used as an alternative to synthetic antioxidants with a higher safety profile [108]. In addition to essential oil, the *O. basilicum* L. hexane extracts also showed possible antioxidant activity in a concentration- and dose-dependent manner [118].

The *O. basilicum* L. in Jordan was tested against three cancer cell lines for anticancer activity, such as the triple-negative breast cancer cell line (MDA-MB-231) with IC_50_ 432.3 ± 32.2 µg/mL, the ER+ breast cancer cell line (MCF7) with IC_50_ 320.4 ± 23.2 µg/mL, and the glioblastoma cancer cell line (U-87 MG) with IC_50_ 431.2 ± 15.3 µg/mL. It turned out that the essential oils containing major components of linalool, eugenol, and eucalyptol exhibited potential anticancer activity [119]. The essential oil was also tested on cancer cell lines from liver cancer (Hep 3B) and breast cancer (MCF-7), which resulted in good cytotoxic effect on both cell lines [115]. In addition to the essential oils, methanolic extracts from the aerial parts of the plant also exhibited promising anticancer activity against MCF-7 and MDA-MB-231. The anticancer properties are shown by the expression level of apoptosis-related genes, which decreases the *bcl-2* gene’s ability to act as an inhibitor protein for programmed cell death and allows the cell to undergo apoptosis. In this study, the anticancer activity of *O. basilicum* L. came from the active compound eugenol [120].

The combination of *Morus nigra* and *O. basilicum* L. extracts was tested in various cancer cell lines and normal human cells for anticancer activity. It was discovered that the chloroform extracts (MO2C) possessed the highest anticancer activity. The reason for this is that MO2C was cytotoxic against all tested cell lines at the lowest concentration—particularly the breast cancer cell line—and had selective cytotoxicity toward the normal cell line. In addition, this extract contains α-*trans*-bergamotene, germacrene D, selin-4,7(11)-diene, 2-decel-1-ol, and 2-tridecen-1-ol, which play anticancer roles. The anticancer capacities observed in cell morphology include shrinkage, loss of cellular integrity, cell detachment, and contraction of the cytoplasm [121].

Another in vitro study demonstrated that the methanolic, hexane, and dichloromethane extracts showed a potential antidiabetic property. These three extracts were subjected to a cytotoxic assay, which is considered safe for methanolic extracts up to 0.25 mg/mL and for hexane and dichloromethane extracts up to 0.5 mg/mL. The hexane extract demonstrated an “insulin-like” effect in the absence of insulin due to translocation of the glucose transporter (GLUT4) to the plasma membrane. This study stated that there were 17 newly identified compounds, which possibly played antidiabetic roles in the extracts. Some of these compounds contained glycerol, cyanuric acid, talose, oleamide, inositol, hydroquinone-beta-d-glucopyranoside, pentane-1,2,5-triol, and glucopyranose. Inositol was first found in the *O. basilicum* L. methanolic extract in this study [122].

Meanwhile, an in vivo study performed in male albino mice showed that *O. basilicum* L. leaf extract administered orally had the potential to improve neuromuscular coordination, active behavior, the ability to recognize objects, and short-term memory. The optimum daily supplementation dose was found to be 100 mg/mL solvent/kg body weight and was considered suitable for oral administration without any safety concerns [123]. Hydroethanolic extract, ethyl acetate, and n-hexane fractions had anticonvulsant and neuroprotective characteristics, which prevented oxidative damage to the brain tissue, with optimum dose at 200 mg/kg [124]. For the first time, new compounds called 5,7-dihydroxy-3′,4′,5′-trimethoxyflavone and 3-hydroxy-3′,4′,5′-trimethoxyflavone have been found in *O. basilicum* L. leaf extracts and fractions. An in silico study showed that both compounds had binding interaction energy for −9.93309 and −15.9683, respectively, with Caspase-3 target protein. Both of these compounds helped improve long-term memory by reducing Caspase-3 concentration and suggesting the role of anti-apoptotic cells against neuron cells. This neuroprotective ability is due to the combination of anticholinergic, antioxidant, anti-inflammatory, and anti-apoptotic effects of the compound [125]. The inhalation of essential oil derived from *O. basilicum* L. has been shown to possess neuroprotective properties and exhibit depressive effects in mice. The essential oil demonstrated antidepressant properties in mice subjected to chronic unpredictable mild stress [126].

Moreover, *O. basilicum* L. has been identified as an effective agent in exerting anti-inflammatory effects. An in vivo study performed in mice showed that the essential oil of *O. basilicum* L. with the estragole (methyl chavicol) chemotype in doses of 100 mg/kg and 50 mg/kg greatly reduced paw edema induced by carrageenan by 74% and 44%, respectively, between the first and fifth hour of evaluation. Furthermore, these doses of essential oil are deemed safe for oral administration [127]. An in vitro study showed that *O. basilicum* L. treated with chemical elicitors, such as arachidonic acid, jasmonic acid, and β-aminobutyric acid, enhanced the flavonoid and phenolic content, which possess anti-inflammatory properties. This finding showed that a plant with arachidonic acid elicitor had the greatest inhibitory effect against lipoxygenase (LOX) (EC_50_ = 1.67 mg FW mL^−1^) and cyclooxygenase (COX) (EC_50_ = 0.31 mg FW mL^−1^). The inhibitory efficacy exhibited positive correlation with the increased content of rosmarinic, benzoic, and *o*-coumaric acids [128]. Moreover, an in vitro study demonstrated that ethanolic leaf and leaf callus extracts significantly reduced nitric oxide as pro-inflammatory mediators with concentrations of 0.01–1 mg/mL on RAW 264.7 macrophage cells. This anti-inflammatory activity may be a result of the major compounds found in the extracts, which are 2,3-dihydroxy-3,5-dihydroxy-6-methyl-4H-pyran-4-one and 2-methoxy-4-vinylphenol [129].

Another in vivo study demonstrated that *O. basilicum* L. methanolic extract emulgel formulation represented a potential alternative for second-degree-burn wound-induced rabbits. Formulations with 5% extract, polymer, and other excipients were compatible and had a good safety profile for topical emulgel. This extract formulation showed a healing capacity on the 16th day, with 98.78% wound contraction, which was insignificantly different to the healing capacity of commercial healing products [130].

In addition, a novel hydrogel formulation based on *O. basilicum* L. and *Trifolium pratense* extract combination had a great wound healing ability. In vitro tests demonstrated that the combined extract with concentration of 50 µg/mL had the greatest healing efficacy in terms of complete healing time and fibroblast density. An in vivo study also showed that the combined extract healed the wound 100% on the 13th day—better than the control group—which means that it exhibited a remarkable wound healing capability. Moreover, the hydrogel formulation was tested in a clinical case of a patient with *Psoriasis vulgaris* twice a day. The formulation was shown to reduce erythema symptoms within one week of treatment. The tremendous wound healing capability could be explained by the synergistic effect of the extract’s phytochemical mixture. The *O. basilicum* L. extract was rich in phenolic and flavonoid contents, especially ferulic acid; meanwhile, the *T. pratense* was rich in chlorogenic acid [131]. Ferulic acid is known for its ability to enhance wound healing through the promotion of angiogenesis, reduction in oxidative stress, and inhibition of bacterial growth [132].

Moreover, there are three novel compounds found in *O. basilicum* L., such as inositol, 5,7-dihydroxy-3′,4′,5′-trimethoxyflavone, and 3-hydroxy-3′,4′,5′-trimethoxyflavone. The studies above indicated that *O. basilicum* L. holds significant potential for development and formulation into a natural pharmaceutical alternative. Thus, additional investigation of the drug delivery system and clinical trials are necessary in order to create natural accessible medication.

## 7. Biotechnological Development in *O. basilicum* L. Research

The field of biotechnology involves the utilization of scientific methodologies to alter and enhance the characteristics of plants, animals, and micro-organisms in order to increase their overall value [133]. The demand for herbal medicine on a global scale is substantial and exhibits a consistent growth rate. Various technologies have been implemented to facilitate the promotion of bioactive compounds in medicinal plants [134]. Secondary metabolites, which are considered vital constituents of the plants, hold significant economic value due to their applications as pharmaceutical products, perfumes, pigments, and food additive products [135].

### 7.1. Green Nanotechnology Production in O. basilicum *L.* for Medical Application

Previously, we discussed the antibacterial and antifungal properties of *O. basilicum* L., which were extensively explored from 2010 to 2018, revealing its potential for combating various bacterial and fungal infections. Over the last five years, numerous studies on nanotechnology have demonstrated the ways of enhancing the antimicrobial properties of this plant. One notable advantage of using plant extracts for synthesizing nanoparticles is their ability to generate a larger zone of inhibition compared to chemical synthesis methods [136].

The essential oil of *O. basilicum* L. had moderate antibacterial activity against Gram-negative bacteria. However, combining and formulating the essential oil into chitosan nanocarriers with nanoencapsulation technology exhibited strong antibacterial and antibiofilm properties against *E. coli* and *S. aureus*, resulting in inhibitory zones measuring 15.3 mm and 21 mm, respectively. This combination damages the cell membrane, and therefore, it causes the leakage of biological macromolecules [137]. Therefore, the combination has good potential for overcoming Gram-negative resistance against antibiotics. ZnO NP is one of the nanoparticles, which showed great antibacterial activity against *Pseudomonas aeruginosa,* with 20 mm inhibitory zone diameter [138].

ZnO NP synthesized with the *O. basilicum* L. extract was tested against other bacteria species and exhibited a great inhibitory zone diameter for *S. aureus* (19.3 mm), *E. coli* (13.2 mm), *S. typhimurium* (8.2 mm), *L. monocytogenes* (11.4 mm), *B. subtilis* (9.3 mm)*,* and *P. aeruginosa* (12.4 mm). It also showed great MIC for antibacterial activity, ranging from 0.78 µg/mL, 1.56 µg/mL, 3.12 µg/mL to 6.25 µg/mL [136]. Along with ZnO NP, copper oxide nanoparticles (CuO NPs) enhance the antibacterial activity against *S. aureus* and *E. coli* more than the extract itself [139]. In addition to the monometallic synthesized nanoparticle, there are bimetallic synthesized nanoparticles. This is a combination of two different types of metallic nanoparticles in one particle, which work synergistically [140]. In this study, a combination of silver and platinum nanoparticles (AgPt NP) exhibited a significant inhibitory effect on *S. aureus*, *E. faecalis*, *E. coli*, and *K. pneumoniae* rather than the monometallic nanoparticle. The bimetallic particle showed an inhibitory diameter of 9–25 mm, whereas the monometallic particle of each nanoparticle only showed an inhibitory diameter under 10 mm [141].

Another study demonstrated the green synthesis of reduced graphene oxide (RGO)-zinc oxide (ZnO) nanocomposite, or RGO-ZnO NCs. It was shown that at a concentration of 30 µg/mL, an inhibition zone was observed for the *Cocci* strain and *E. coli* at 20 mm and 10 mm, respectively. RGO-ZnO NCs had antibacterial activity at a small concentration, whereas the essential oil or extract of *O. basilicum* L. itself needed higher concentrations to achieve the same results. This study will also become the basis for the next development and investigation of RGO-ZnO NCs as potential antioxidant candidates and diabetes treatments [142]. Another potential diabetic therapy based on a synthesized silver nanoparticle was found in *O. basilicum* L. leaf extract. The result demonstrated inhibitory activity against α-amylase—which was higher than antidiabetic medicine acarbose—and high inhibitory activity against α-glucosidase, higher than acarbose and crude extract [143]. This finding suggests the need for alternative therapies for diabetic treatment.

A recent study demonstrated that *O. basilicum* L. chemical constituents were responsible for the green biosynthesis of ZnO NPs. In combination with bacterial phages, ZnO NPs demonstrated antibacterial activity against *Salmonella enterica* and deformation on biofilm, which were caused by *Staphylococcus sciuri* [144]. Another study demonstrated the green synthesis of silver nanoparticles (Ag NPs) in combination with phage ZCSE6 for antibacterial activity against *Salmonella enterica*. The *O. basilicum* L. extract works as a bio-reducing agent in order to create Ag NPs effectively. It was shown that the Ag NPs exhibited antibacterial activity; the minimum concentration to inhibit growth was 6.25 µg/mL, and the minimum bactericidal concentration was 12.5 µg/mL. Surprisingly, the Ag NPs in combination with phage ZCSE6 had great bactericidal activity, with a lower concentration than the MIC, which suppressed the growth of *S. enterica* 24 h after treatment [145].

In addition to utilization of the *O. basilicum* L. extract for synthesizing nanoparticles, the mucilage from the seed in combination with nanoparticles can create a novel natural wound dressing. Basil seed mucilage (BSM) was dried and then combined with ZnO NP to create a hydrogel sponge. As the weight percent (wt%) of ZnO NP increased, the antibacterial activity of the BSM hydrogel sponge was enhanced. It exhibited great antibacterial activity at 50 wt% ZnO NP against *E. coli* and *S. aureus*, with an inhibitory zone at 15.9 mm and 16.7 mm, respectively. The increasing ZnO NP wt% content on the hydrogel sponge also resulted in a slight decrease in thickness, porosity, degree of swelling, and a slight increase in the water holding capacity. The BSM with ZnO NP is considered non-toxic to human keratinocyte (HaCat) cells [146]. This hydrogel sponge could have the potential to be commercialized as a natural healthcare product.

In addition to antibacterial functions against human pathogens, the synthesis of silver nanoparticles (Ag NPs) can also work as a control agent for the management of plant viral infections. This study tested Ag NPs against cucumber mosaic virus (CMV), which infects squash. Spraying the foliar containing Ag NPs at a concentration of 100 µg/mL resulted in enhanced growth, delayed indication of disease symptoms, and a significant reduction of up to 92% in CMV accumulation levels as compared to the non-treated plants. It also increased the soluble carbohydrate, free radical scavenging activity, antioxidant enzymes, and total phenolic and flavonoid contents. This finding could be an alternative for treating plant viral disease instead of using chemical biocides [147]. There is a substantial opportunity in developing a green synthesis of nanoparticles within the *O. basilicum* L. extracts, which could be a potential therapy and alternative treatment in many cases of human diseases.

### 7.2. Biotechnological Techniques for Improving the Metabolite Production of O. basilicum *L.*

It is known that the medicinal plant *O. basilicum* L. is a rich source of valuable phytoconstituents [148]. The diversity of chemical compounds in *O. basilicum* L., alone or in synergy, exhibits some medicinal properties [149]. The current production of horticulture crops is centered on improving the quality, quantity, and safety of products, as well as yield, in order to meet the demands of the food and health industries, which have a strong reliance on chemical compounds [150]. The advancement of *O. basilicum* L. production is influenced by various aspects, including environmental parameters (light, soil nutrients, temperature, water, CO_2_ levels), cultivars, and cultivation methods [151]. Several studies have demonstrated various experiments on how to improve the chemical compounds derived from the plant *O. basilicum* L.

One study showed that narrow-bandwidth light treatments of basil seeds were observed to have relative effects on volatile oils. Light conditions may increase the value and quality of this herb, which is appreciated for human wellness. Light treatments could induce the three main compounds in *O. basilicum* L., which are eugenol, linalool, and 1,8-cineol (eucalyptol). Eugenol and linalool are induced by blue-red-green (BRG) light, and 1,8-cineole is induced by BRG, blue-red-yellow (BRY), and blue-red-far-red (BRFr) light [152]. These compounds mainly play a role as antimicrobial and antioxidant agents [153]. The blue and red LED treatments can potentially improve *O. basilicum* L. growth and increase the phenolic content of the plants; thus, the different cultivars can also have a different result. The green cultivar in this study was mostly stimulated by the red light, and the red cultivar was stimulated by the blue light [154].

In addition to the light treatments, the abiotic (CdCl_2_ and AgNO_3_) and biotic (YE) yeast extract elicitors were found to increase the total amount of phenolic and flavonoid contents. Chicoric and rosmarinic acid increased with the treatment of CdCl_2_ and AgNO_3_ at 5 µM. Rutin and isoquercetin also increased with the YE treatment, up to 1.6 times and 1.9 times. Meanwhile, the highest amounts of linalool and estragole were observed in the treatment with AgNO_3_, up to 2.8 times and 0.5 times [155]. Arbuscular mycorrhizal fungi (AMF), which are another type of biotic elicitors, showed a promising capacity in increasing the production of essential oil, with eugenol and γ-cadinene being the compounds with the highest ratios, which composed the essential oil [156].

Various methods of enhancing the chemical compounds of *O. basilicum* L. were discussed above to emphasize the importance of naturally synthesized compounds. One such approach involves improving the growth factors through light treatments and optimizing the formulation of biotic or abiotic elicitors.

## 8. Conclusions

*O. basilicum* L. is a plant species, which exhibits wide distribution throughout several regions of the world. Over time, there has been a significant evolution in the understanding and application of this plant in the context of healthcare. The plant is regarded as a highly valuable source due to its distinctive chemical composition, which provides a diverse array of antimicrobial and other medicinal attributes, including anticancer, antioxidant, antidiabetic, and neuroprotective functions. This plant could change the way in which drugs are produced, either by isolating pure phytochemical compounds or by combining several compounds. This could revolutionize the pharmaceutical industry by providing a natural substitute for synthetic drugs.

The utilization of *O. basilicum* L. as a medicinal plant has developed over years, starting from community beliefs. The beliefs held by communities were recorded, and many studies were conducted to prove the efficacy of this plant. Several studies have highlighted the potential of the aerial parts—particularly the leaves—of this plant for the development of novel medicines. The phytochemical classes predominantly associated with antimicrobial and biomedical activities are polyphenols, terpenes, and phytosterols. Noteworthy compounds with promising potential for antiviral drug development include carvacrol, α-guaiene, ursolic acid, apigenin, stigmasterol, and campesterol. Additionally, compounds such as linalool, rutin, eugenol, estragole, citral, α-pinene, nerolidol, kaempferol, and trans-α-bergamotene could be utilized in the creation of medicines targeting bacteria and fungi. Moreover, the exploration of new neuroprotective medicines may be facilitated by novel compounds, such as 5,7-dihydroxy-3′,4′,5′-trimethoxyflavone and 3-hydroxy-3′,4′,5′-trimethoxyflavone. Furthermore, topical formulation for wound healing has been demonstrated to be a promising alternative treatment.

Furthermore, evidence is becoming the key point to be subsequently developed and formulated into a novel drug. In terms of the plant’s usefulness in the healthcare field, its distinctive chemical compound, and its higher safety profile, future research should focus more on formulating this plant into a natural choice in addition to chemical drugs. As another alternative, this plant could be combined with chemical drugs to create new efficacy and new mechanisms, which are destined for later commercialization.

Future innovations could come from researching the ways to patent the extraction methods, formulating a standardized approach to boost the chemical compound contents, conducting more clinical research in a mechanistic and molecular way, and advancing it up to the industrial stage. This will allow not only gaining a deeper understanding of the mechanistic action, but it will also lead the path to developing more effective, safer drugs and reduce undesirable side effects.

## Figures and Tables

**Table 1:** Chemical constituents from extracts and essential oil of *O. basilicum* L.

No.	Chemical Compound	Molecular Weight	Source	Extraction and Identification Method	Reference
	**Monoterpene Hydrocarbon**				
1.	α-pinene	136.23 g/mol	Leaf	Hydrodistillation, GC-MS Maceration 24 h, GC-MS Hydrodistillation, solvent extraction, GC-MS	[34] [35] [36]
2.	β-Myrcene	136.23 g/mol	Leaf	Hydrodistillation, GC-MS	[34]
3.	Citral	153.23 g/mol	Leaf	Hydrodistillation, GC-MS	[34]
4.	Camphene	136.23 g/mol	Leaf	Maceration, GC-MS SFME, hydrodistillation 1 h, GC-MS	[32] [37]
5.	Terpineol	154.25 g/mol	Leaf	Maceration, GC-MS	[32]
6.	Linalyl acetate	196.29 g/mol	Leaf	Maceration, GC-MS	[32]
7.	*cis*-Sabinene hydrate	154.25 g/mol	Leaf	Maceration, GC-MS	[32]
8.	(−)-*trans*-Pinocarvyl acetate	194.27 g/mol	Leaf	Maceration, GC-MS	[32]
9.	Limonene dioxide	168.23 g/mol	Leaf	Maceration, GC-MS Hydrodistillation, GC-MS	[32] [38]
10.	Geraniol	154.25 g/mol	Leaf	Hydrodistillation, GC-MS	[38]
11.	Carvone	150.22 g/mol	Leaf	Hydrodistillation 3 h, GC-MS	[39]
12.	Myrtenol	152.23 g/mol	Leaf	Hydrodistillation, GC-MS	[40]
13.	Fenchone	152.23 g/mol	Aerial parts	Maceration 24 h, GC-MS Hydrodistillation 4 h, GC-MS	[35] [28]
	**Oxygenated Monoterpene**				
14.	l-Menthol	156.26 g/mol	Leaf	Hydrodistillation, GC-MS	[34]
15.	Linalool	154.25 g/mol	Leaf	Maceration, GC-MS Hydrodistillation, GC-MS	[32] [38]
16.	*trans*-linalool oxide	170.25 g/mol	Leaf	Maceration, GC-MS	[32]
17.	cis-Linalool-oxide	213.27 g/mol	Leaf	Hydrodistillation, GC-MS Maceration, GC-MS	[34] [32]
18.	Camphor	152.23 g/mol	Leaf	Maceration, GC-MS Maceration 24 h, GC-MS SFME, hydrodistillation 1 h, GC-MS	[32] [35] [37]
19.	Neral	152.23 g/mol	Aerial parts	Maceration 24 h, GC-MS SFME, hydrodistillation 1 h, GC-MS	[35] [37]
20.	1,8-cineole (Eucalyptol)	154.25 g/mol	Leaf	Hydrodistillation, GC-MS Maceration, GC-MS Hydrodistillation, solvent extraction, GC-MS	[34] [32] [36]
21.	Estragole (Methyl chavicol)	148.20 g/mol	Leaf, flower, inflorescence	Hydrodistillation, GC-MS Hydrodistillation 3 h, GC-MS Maceration 24 h, GC-MS Hydrodistillation, solvent extraction, GC-MS	[34] [39] [35] [36]
22.	Eugenol	164.20 g/mol	Leaf, aerial parts	Hydrodistillation, GC-MS Maceration 24 h, GC-MS SFME, hydrodistillation 1 h, GC-MS	[34] [35] [37]
23.	Methyl eugenol	173.23 g/mol	Leaf	Hydrodistillation 3 h, GC-MS Maceration 24 h, GC-MS	[39] [35]
24.	Bornyl acetate	196.29 g/mol	Aerial parts	Hydrodistillation 4 h, GC-MS	[28]
25.	Methyl cinnamate	162.18 g/mol	Leaf	Hydrodistillation, GC-MS	[40]
	**Sesquiterpene Hydrocarbon**				
26.	Copaene	204.35 g/mol	Leaf	Hydrodistillation, GC-MS	[34]
27.	Neoisolongifolene	202.33 g/mol	Leaf	Hydrodistillation, GC-MS	[34]
28.	α-Bergamotene	204.35 g/mol	Leaf	SFME, hydrodistillation 1 h, GC-MS	[37]
29.	*trans*-.alpha.-Bergamotene	204.35 g/mol	Leaf	Hydrodistillation, GC-MS Hydrodistillation, solvent extraction, GC-MS Hydrodistillation, GC-MS	[34] [36] [38]
30.	β-farnesene	204.35 g/mol	Leaf	Hydrodistillation, GC-MS	[38]
31.	Alloaromadendrene	204.35 g/mol	Leaf	Hydrodistillation, GC-MS	[34]
32.	γ-Cadinene	204.35 g/mol	Leaf	SFME, hydrodistillation 1 h, GC-MS	[37]
33.	Humulene	204.35 g/mol	Leaf	Hydrodistillation, GC-MS	[34]
34.	α-Humulene	204.35 g/mol	Leaf	SFME, hydrodistillation 1 h, GC-MS	[37]
35.	α-Copaene	204.35 g/mol	Leaf	SFME, hydrodistillation 1 h, GC-MS	[37]
36.	β-Copaene	204.35 g/mol	Leaf	Hydrodistillation, GC-MS	[34]
37.	β-Bisabolene	204.35 g/mol	Leaf	Hydrodistillation, GC-MS	[34]
38.	cis-muurola-3,5-diene	204.35 g/mol	Leaf	Hydrodistillation, GC-MS	[34]
39.	cis-.alpha.-Bisabolene	204.35 g/mol	Leaf	Hydrodistillation, GC-MS	[34]
40.	α-Cubebene	204.35 g/mol	Leaf, aerial parts	Hydrodistillation, GC-MS Maceration 24 h, GC-MS Hydrodistillation, solvent extraction, GC-MS	[40] [35] [36]
41.	Germacrene B	204.35 g/mol	Leaf	Hydrodistillation, GC-MS	[40]
42.	Germacrene D	204.35 g/mol	Leaf	Hydrodistillation, GC-MS Hydrodistillation 4 h, GC-MS Maceration 24 h, GC-MS Hydrodistillation, GC-MS	[40] [28] [35] [38]
43.	β-Elemene	204.35 g/mol	Leaf	SFME, hydrodistillation 1 h, GC-MS	[37]
44.	β-Cubebene	204.35 g/mol	Aerial parts	Maceration 24 h, GC-MS Hydrodistillation, solvent extraction, GC-MS	[35] [36]
45.	β-Caryophyllene	204.35 g/mol	Aerial parts	Maceration 24 h, GC-MS Hydrodistillation, GC-MS SFME, hydrodistillation 1 h, GC-MS	[35] [38] [37]
	**Oxygenated Sesquiterpene**				
46.	α-Bisabolol	222.37 g/mol	Aerial parts	Hydrodistillation 4 h, GC-MS	[28]
47.	α-Cadinol	222.37 g/mol	Aerial parts	Hydrodistillation 4 h, GC-MS	[28]
48.	Nerolidol	222.37 g/mol	Leaf	SFME, hydrodistillation 1 h, GC-MS	[37]
49.	Caryophyllene oxide	220.35 g/mol	Aerial parts	Maceration 24 h, GC-MS	[35]
	**Other Compounds**				
50.	*trans*-4-Methoxycinnamaldehyde	162.18 g/mol	Leaf	Hydrodistillation, GC-MS	[34]
51.	Mandelic Acid (Benzeneacetic acid, alpha.-hydroxy)	152.15 g/mol	Leaf	Hydrodistillation, GC-MS	[34]
52.	Phenylethanolamine	137.18 g/mol	Leaf	Hydrodistillation, GC-MS	[34]
53.	N-Benzyl-N-ethyl-p-isopropylbenzamide	281.4 g/mol	Leaf	Hydrodistillation, GC-MS	[34]
54.	*cis*-2-(2-pentenyl) furan	136.19 g/mol	Leaf	Maceration, GC-MS	[32]

SFME: Solvent-free microwave extraction.

**Table 2:** Minimum inhibitory concentration (MIC) value of antibacterial activity of *O. basilicum* L.

Bacterial Species	Essential Oil/Extract	MIC Value	Reference
**Gram Positive**			
*Bacillus cereus* (ATCC 11778)	Essential oil Essential oil and methanolic extract	10.80 µL/mL 62.5 µg/mL	[92] [93]
*Bacillus subtilis*	Essential oil and methanolic extract	125 µg/mL	[93]
*Bacillus megaterium*	Methanolic extract	62.5 µg/mL	[93]
*Enterococcus faecalis* (ATCC 19433)	Essential oil	0.75 mg/mL	[94]
*Listeria monocytogenes*	Essential oil and methanolic extract	125 µg/mL	[93]
*Micrococcus luteus* (ATCC 10240)	Essential oil	0.50 mg/mL	[94]
*Sarcina* sp.	Essential oil	0.75 mg/mL	[94]
*Staphylococcus aureus* (ATCC 6538P)	Essential oil Essential oil Essential oil Essential oil and methanolic extract	2.45 µL/mL 32 µg/mL 1 mg/mL 62.5 µg/mL	[92] [95] [94] [93]
*Staphylococcus epidermidis*	Essential oil	0.75 mg/mL	[94]
*Streptococcus mutans*	Essential oil	0.75 mg/mL	[94]
**Gram Negative**			
*Acinetobacter* sp.	Essential oil	0.75 mg/mL	[94]
*Aeromonas* sp.	Essential oil	1 mg/mL	[94]
*Citrobacter freundii* (ATCC 8090)	Essential oil	1 mg/mL	[94]
*Escherichia coli* (ATCC 25922)	Essential oil Methanolic extract	10.80 µL/mL 125 µg/mL	[92] [93]
*Klebsiella pneumoniae* (ATCC 13833)	Essential oil	0.75 mg/mL	[94]
*Proteus mirabilis* (ATCC 25933)	Essential oil	1 mg/mL	[94]
*P. vulgaris* (ATCC 13315)	Essential oil	0.75 mg/mL	[94]
*Pseudomonas aeruginosa* (ATCC 27853) *P. aeruginosa* (ATCC 25853) *P. aeruginosa* (1662339)	Essential oil Essential oil Essential oil	22.68 µL/mL 256 µg/mL 32 µg/mL	[92] [95] [95]
*Salmonella choleraesuis* (ATCC 10708)	Essential oil	0.5 mg/mL	[94]
*Salmonella typhimurium* (ATCC 14028)	Essential oil	22.68 µL/mL	[92]
*Serratia marcescens* (ATCC 13880)	Essential oil	0.25 mg/mL	[94]
*Shigella boydii*	Essential oil	250 µg/mL	[93]
*Shigella dysenteriae*	Essential oil and methanolic extract	250 µg/mL	[93]
*Shigella flexneri* (ATCC 12022)	Essential oil	0.75 mg/mL	[94]
*Vibrio parahaemolyticus*	Essential oil	250 µg/mL	[93]
*Vibrio mimicus*	Essential oil	250 µg/mL	[93]
*Yersinia enterocolitica* (ATCC 10460)	Essential oil	0.25 mg/mL	[94]

**Table 3:** Diameter of zone inhibition of antibacterial activity of *O. basilicum* L.

Bacterial Species	Essential Oil/Extract	Diameter of Zone Inhibition	Reference
**Gram Positive**			
*Bacillus cereus*	Essential oil Ethyl acetate fraction	25 mm 21.1 mm	[96] [93]
*Bacillus subtilis*	Ethyl acetate fraction Methanolic extract	19.3 mm 31.86 mm	[93] [97]
*Bacillus megaterium*	Ethyl acetate fraction	18.2 mm	[93]
*Clostridium perfringens* type C	Methanolic extract	31.13 mm	[97]
*Cutibacterium acnes* (ATCC 11827)	Essential oil	18.13 mm	[38]
*Enterococcus* sp.	Methanolic extract	30.73 mm	[97]
*Enterococcus faecalis* (ATCC 19433)	Essential oil Essential oil	10.3 mm 11.2 mm	[94] [98]
*Listeria monocytogenes*	Essential oil	17.1 mm	[93]
*Micrococcus luteus* (ATCC 10240)	Essential oil	13.5 mm	[94]
*Sarcina* sp.	Essential oil	14.6 mm	[94]
*Staphylococcus aureus* (ATCC 6538) *S. aureus* (ATCC 6538) *S. aureus* (ATCC 25923) *S. aureus*	Essential oil Ethyl acetate fraction Essential oil Methanolic extract	9 mm 17.1 mm 9.7 mm 30.66 mm	[96] [93] [98] [97]
*Staphylococcus epidermidis* (ATCC 12228)	Essential oil	13.3 mm	[98]
*Staphylococcus mutans* (ATCC 25175)	Essential oil	11 mm	[94]
**Gram Negative**			
*Acinetobacter* sp.	Essential oil	15 mm	[94]
*Aeromonas* sp.	Essential oil	10.6 mm	[94]
*Citrobacter freundii* (ATCC 8090)	Essential oil	11.6 mm	[94]
*Escherichia coli* *E. coli* *E. coli* *E. coli* *E. coli* (ATCC 25922)	Essential oil Essential oil Ethyl acetate fraction Methanolic extract Essential oil	11 mm 10.3 mm 14.2 mm 28.30 mm 13.5 mm	[96] [94] [93] [97] [98]
*Klebsiella pneumoniae*	Essential oil Essential oil Methanolic extract	12.2 mm 17.2 mm 26.66 mm	[94] [98] [97]
*Proteus mirabilis* (ATCC 25933)	Essential oil Essential oil	11.3 mm 13.1 mm	[94] [98]
*Proteus vulgaris* (ATCC 13315)	Essential oil	18 mm	[94]
*Pseudomonas aeruginosa*	Methanolic extract	28.83 mm	[97]
*Salmonella choleraesuis* (ATCC 10708)	Essential oil	10 mm	[94]
*Salmonella typhymurium*	Essential oil Methanolic extract	10 mm 15.30 mm	[96] [97]
*Serratia marcescens* (ATCC 13880)	Essential oil Essential oil	16.6 mm 10.4 mm	[94] [98]
*Shigella boydii*	Essential oil	13.3 mm	[93]
*Shigella dysenteriae*	Ethyl acetate fraction	15.2 mm	[93]
*Shigella flexneri* (ATCC 12022)	Essential oil	17.1 mm	[94]
*Vibrio parahaemolyticus*	Ethyl acetate fraction	16.2 mm	[93]
*Vibrio mimicus*	Methanolic extract	51.2 mm	[93]
*Xanthomonas* sp.	Methanolic extract	14.36 mm	[97]
*Yersinia enterocolitica* (ATCC 10460)	Essential oil	12.6 mm	[94]

## Data Availability

This study did not report any data.
